# Effect of L-Carnitine on Skeletal Muscle Lipids and Oxidative Stress in Rats Fed High-Fructose Diet

**DOI:** 10.1155/2007/72741

**Published:** 2007-04-12

**Authors:** Panchamoorthy Rajasekar, Carani Venkatraman Anuradha

**Affiliations:** Department of Biochemistry and Biotechnology, Faculty of Science, Annamalai University, Annamalai Nagar 608 002, India

## Abstract

There is evidence that high-fructose diet induces insulin resistance, alterations in lipid metabolism, and oxidative stress in rat tissues. The purpose of this study was to evaluate the effect of L-carnitine (CAR) on lipid accumulation and peroxidative damage in skeletal muscle of rats fed high-fructose diet. Fructose-fed animals (60 g/100 g diet) displayed decreased glucose/insulin (G/I) ratio and insulin sensitivity index (ISI_0,120_) indicating the development of insulin resistance. Rats showed alterations in the levels of triglycerides, free fatty acids, cholesterol, and phospholipids in skeletal muscle. The condition was associated with oxidative stress as evidenced by the accumulation of lipid peroxidation products, protein carbonyls, and aldehydes along with depletion of both enzymic and nonenzymic antioxidants. Simultaneous intraperitoneal administration of CAR (300 mg/kg/day) to fructose-fed rats alleviated the effects of fructose. These rats showed near-normal levels of the parameters studied. The effects of CAR in this model suggest that CAR supplementation may have some benefits in patients suffering from insulin resistance.

## 1. INTRODUCTION

Rats fed a high-fructose diet form a model of diet-induced insulin resistance. The condition is associated with hyperinsulinemia, hypertriglyceridemia, and glucose intolerance [1]. The metabolic effects are similar to those observed in the human multimetabolic syndrome, or syndrome X, in which a cluster of disorders such as insulin resistance, hypertension, dyslipidemia, and glucose intolerance are described [2]. High fructose diet has prooxidant effects. Both enhanced oxidative damage to cellular constituents and diminished antioxidative capacity have been reported in fructose-fed rats [3, 4].

L-carnitine (CAR, *β*-hydroxy-*γ*-trimethylaminobuty-rate), a natural vitamin-like compound, is a ubiquitous constituent of mammalian plasma and tissues, mainly distributed among skeletal and cardiac muscles. CAR is supplied to the body through dietary sources (e.g., meat, dairy products), and by biosynthesis from lysine and methionine. CAR functions to transport long-chain fatty acids across the inner mitochondrial membrane into the matrix for *β*-oxidation and has effects on oxidative metabolism of glucose in tissues [5].

Supplementation studies have shown that CAR promotes insulin sensitivity [6] and has hypolipidaemic actions [7]. Previously we have demonstrated that CAR could improve insulin action in the fructose-fed rat model of insulin resistance [8]. Skeletal muscle is an insulin-sensitive tissue which is also a site of insulin resistance in the fructose-fed rat [9] and is vulnerable to oxidative damage.

Considering all these, this study was initiated to evaluate the role of CAR in mitigating oxidative stress and lipid accumulation in the insulin sensitive skeletal muscle in a well-characterized model of insulin resistance.

## 2. MATERIALS AND METHODS

### 2.1. Chemicals and drugs

L-carnitine (CAR) was obtained from the Sisco Research Laboratories (P) Ltd., Mumbai, India. All other chemicals and solvents were of analytical grade and were purchased from Himedia Laboratories Pvt. Ltd., Mumbai, India.

### 2.2. Animals and treatment

Adult male Wistar rats body weight ranging from 150 g to 160 g were obtained from the Central Animal House, Rajah Muthiah Medical College, Annamalai University. They were housed in an animal room under controlled conditions on a 12 h light/12 h dark cycle. Animals received a standard pellet diet (Karnataka State Agro Corporation Ltd., Agro feeds division, Bangalore, India) and water ad libitum. Insulin resistance was induced by feeding high fructose diet (60 g/100 g). The experimental procedures were approved by the Institutional Ethical Committee of Animal Care, Rajah Muthiah Medical College, Annamalai University.

The animals were divided into four groups of 6 rats each and were maintained as follows.

**Group1**

(CON)—Control animals received the control diet containing starch and tap water ad libitum.

**Group2**

(FRU)—Fructose-fed animals received the fructose-enriched diet and water ad libitum.

**Group3**

(FRU + CAR)—Fructose-fed animals received the fructose diet and were administered CAR (300 mg/Kg/day, intraperitoneally).

**Group4**

(CON + CAR)—Control animals received the control diet and were administered CAR (300 mg/Kg/day, intraperitoneally).

The diet composition is given in Table 1. The animals were maintained in their respective groups for 30 days.

### 2.3. Insulin sensitivity assessment

On day 29, the rats were fasted overnight, blood samples were collected by sinoocular puncture at 0 and 120 minutes after administration of glucose (2 g/Kg). The blood glucose concentration was quantified by glucose oxidase method using kit and plasma insulin was estimated by microparticle enzyme immunoassay method, with a kit obtained from Boehringer Manheim, Germany. Insulin sensitivity was calculated by comparing the fasting glucose/insulin ratio among the experimental groups. The insulin sensitivity index (ISI_0,120_) was calculated [10] using the formula

(1) $${\text{ISI}}_{0,120}=\frac{\text{MCR}}{\log\text{MSI}}$$,

where, metabolic clearance rate

(2) $$\text{MCR}=\frac{\text{m}}{\text{MPG}}$$,

MPG = mean plasma glucose, the mean of 0 and 120min glucose values,

MSI = mean serum insulin (mU/I) calculated as the mean of the 0 and 120 min insulin values,

m = [75, 000 mg + (0 min glucose–120 min glucose) × 0.19 × BW]/120 min.

On day 30, the rats were decapitated and skeletal muscle tissue was removed, cleaned, dried, and processed for biochemical measurements. Tissue homogenate was prepared with 0.1 M Tris-HCl buffer, pH 7.4. Blood and tissue homogenates were used for the following investigations.

### 2.4. Lipid analysis

The extraction of lipids from skeletal muscle was carried out according to the procedure of Folch et al. [11]. Total cholesterol [12], phospholipids [13], triglycerides (TG) [14], and free fatty acids [15] were analysed.

### 2.5. Oxidative stress markers and antioxidants

The content of thiobarbituric acid reactive substances (TBARS), lipid hydroperoxide (LHP) and conjugated dienes (CD) were measured by the methods described elsewhere [16]. For TBARS measurement, tissue homogenate was deprotenized with 10% trichloroacetic acid (TCA) and the precipitate was treated with thiobarbituric acid (TBA) at 90^°^C for 1 hour. The pink color formed gave a measure of TBARS. 1,1′,3,3′-tetra methoxy propane was used as the standard and the concentration was expressed as *μ*mol/mg protein.

LHP content was measured in methanol-extracted tissue homogenates. A 0.2 mL aliquot of lipid sample was mixed with 1.8 mL of the reagent, which contained 90 mL of methanol, 10 mL of 250 mM sulphuric acid, 88 mg of butylated hydroxytoluene, 7.6 mg of xylenol orange, and 9.8 mg of ferrous ammonium sulphate. The color developed was read at 560 nm.

For measuring CD, lipids were extracted from skeletal muscle using chloroform/methanol (2 : 1) mixture. Aliquots of lipid extract were evaporated to dryness and suspended in 5.0 mL of methanol, and the ratio of absorbance at 233 nm to that at 215 nm (A_233_/A_215_) was computed. This reflected the extent of peroxidation in the lipid sample.

The level of protein carbonyl was measured by the method of Levine et al. [17]. The tissue was homogenized in 10 mM HEPES buffer containing 137 mM NaCl, 4.6 mM potassium chloride, 1.0 mM potassium dihydrogen phosphate, and 0.6 mM magnesium sulphate. The homogenate was centrifuged at 40 000 g for 20 minutes. The supernatant was mixed with dinitrophenyl hydrazine (DNPH) in 2 N hydrochloric acid and allowed to stand at room temperature for 1 hour. The protein-hydrazone derivative was precipitated with TCA and the precipitate was washed three times with ethanol-ethylacetate (1 : 1). The color in the supernatant was read at 390 nm.

The concentration of aldehydes in skeletal muscle was measured by a fluorescence method [18]. Aliquots of 1 mL of homogenate were extracted with 6 mL of choloform-methanol (2 : 1) and vortexed. The extract was mixed with 6 mL of water and centrifuged for 5 minutes at 3 000 g. To 2 mL of the chloroform layer, 0.2 mL of methanol was added and the fluorescence intensity of this solution was measured at an excitation wavelength of 360 nm and an emission wavelength of 430 nm, using a Perkin-Elmer 512 double beam fluorescent spectrophotometer. Quinine sulphate (0.1 *μ*g/mL) 0.1 M in sulphuric acid (H_2_SO_4_) was used as the standard. The concentration of aldehyde conjugates are given as *μ*mol of quinine sulphate (QS) equivalent/g tissue.

Activities of superoxide dismutase (SOD) (E.C.1.15.1.1), catalase (CAT) (E.C.1.11.1.6), glutathione peroxidase (GPx) (E.C.1.15.1.9), glutathione S-transferase (GST) (E.C.2.5.1.14), and the vitamins C and E were measured in skeletal muscle by methods described elsewhere [16]. Briefly, SOD was assayed by the inhibition of the formation of NADH-phenazine methosulphate nitroblue terazolium formazan. CAT and GPx activities were assayed by measuring the amount of the substrate consumed (hydrogen peroxide and glutathione, resp.) after carrying out the reactions for a specified period of time. GST was assayed using 1-chloro,2,4-dinitrobenzene as a substrate. *α*-Tocopherol was estimated by the reduction of ferric ions to ferrous ions by *α*-tocopherol and the formation of a red-colored complex with 2,2′ dipyridyl was measured at 520 nm. Ascorbic acid was measured by the conversion to dehydroascorbic acid in presence of thiourea, a mild reducing agent and then coupled with DNPH. The compound is converted into a red-colored complex when treated with H_2_SO_4_, which was read at 520 nm.

Total (T-SH), nonprotein (NP-SH) and protein bound (P-SH) sulfhydryl groups were determined by the method of Sedlak and Lindsay [19].

For T-SH measurement, the homogenate in Tris buffer was treated with dithionitrobenzoic acid (DTNB, 99 mg/ 25 mL methanol) and made up to 10 mL with absolute methanol. The mixture was centrifuged at 3000 g for 15 minutes. The absorbance of the clear supernatant was read at 412 nm.

For NP-SH, tissue homogenate was treated with 50% TCA. The thiol content was determined in the supernatant by the reaction with DTNB using glutathione as the standard. P-SH value was obtained by substracting NP-SH from TSH. Protein content was determined by the method of Lowry et al. [20].

### 2.6. Statistical analysis

Values are expressed as means ± SD. Data within the groups are analyzed using one-way analysis of variance followed by Duncan's multiple range test. A value of *P* < .05 was considered statistically significant.

## 3. RESULTS

**Figure 1:**
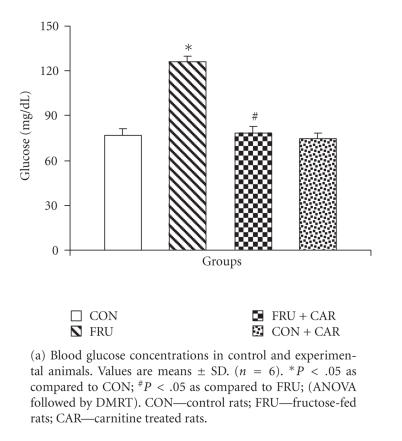

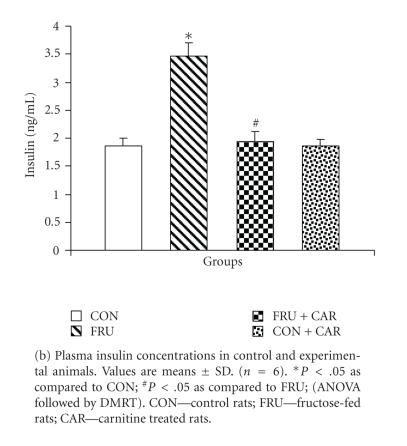

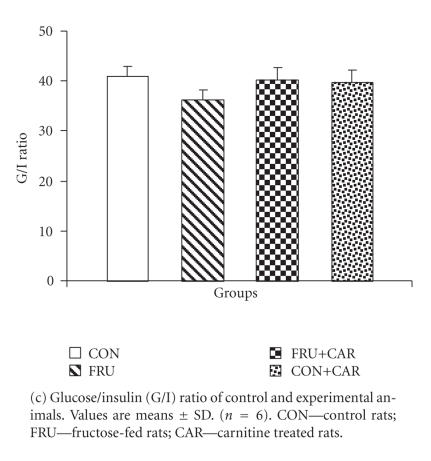

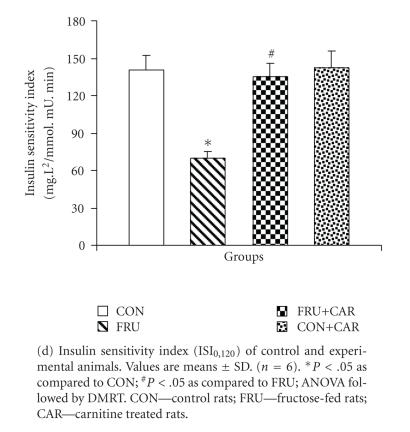


Figures 1(a) and 1(b) show the levels of plasma glucose and insulin, respectively. Figures 1(c) and 1(d) represent G/I ratio and the insulin sensitivity index ISI_0,120_, respectively. The values of glucose and insulin were significantly elevated in FRU as compared to CON while insulin sensitivity index (ISI_0,120_) and glucose/insulin (G/I) ratio were lower. FRU + CAR group registered significantly decreased plasma glucose and insulin levels and increased ISI_0,120_ value and G/I ratio as compared to FRU. The values did not differ significantly between CON and CON + CAR.

**Figure 2:**
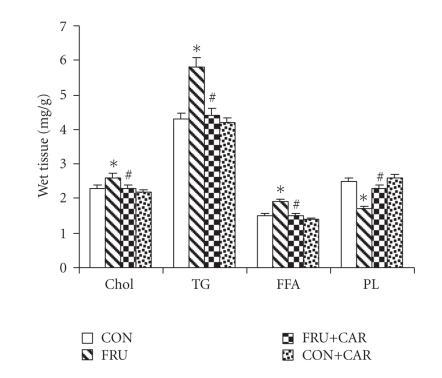
Concentrations of cholesterol, TG, FFA, and PL in skeletal muscle of control and experimental animals. Values are means ± SD. (*n* = 6). ^*^ *P* < .05 as compared to CON; ^#^ *P* < .05 as compared to FRU; ANOVA followed by DMRT. CON—control rats; FRU—fructose-fed rats; CAR—carnitine treated rats. Chol—cholesterol; TG—triglyceride; FFA—free fatty acids; PL—phospholipids.

Concentrations of lipids in skeletal muscle of control and experimental animals are given in Figure 2. The levels of cholesterol, TG, and FFA were significantly increased by 13%, 35%, and 27%, respectively, in FRU as compared to the control-diet fed rats. FRU + CAR rats showed significant decreases (*P* < .05) in cholesterol, TG, and FFA levels as compared to FRU. Phospholipid level was significantly lower (*P* < .05; 32%) in FRU as compared to CON. CAR administration brought the concentrations of lipid constituents to near-normal in FRU + CAR.

Table 2 gives the status of oxidative stress parameters in skeletal muscle of control and experimental animals. FRU groups showed significantly higher levels oxidative stress markers such as LHP, TBARS, CD, and PC associated with accumulation of aldehydes as compared to CON. In FRU + CAR, the levels of these substances were significantly lower (*P* < .05) as compared to FRU.

The antioxidants SOD, CAT, GPx, GST, *α*-tocopherol, ascorbic acid, and thiols were significantly lower in FRU than in CON (Tables 3 and 4). In FRU + CAR, the activities of both enzymatic and nonenzymatic antioxidants were significantly higher as compared to untreated FRU. CON + CAR showed no alterations in the lipid levels and lipid peroxidation indices. We found increased antioxidant levels in these rats which however were not significant.

## 4. DISCUSSION

The development of insulin resistance in fructose-fed rats is well documented in the literature [1, 2] and has been established in our laboratory [8, 16]. Defects in post-receptor events in insulin signaling [21] and in enzymes involved in glucose metabolism [22] have been reported. Fructose feeding decreases the efficacy of insulin extraction by the liver, which retards insulin clearance from the circulation. Further, high intracellular glucose exerts toxic effects on structure and function of organs, and induces insulin resistance, a phenomenon referred to as glucose toxicity. Glucose toxicity is observed in skeletal muscle of diabetic rats [23].

Fructose is a highly lipogenic nutrient. We have earlier reported a rise in cholesterol, TG, and FFA in blood and liver of FRU [24, 25]. Excessive FFA delivery to muscle from the circulation can be a source of muscle TG accumulation. The unregulated fructose metabolism generates both glycerol and acyl portions of acyl-glycerol molecules, the substrates for TG synthesis. Increase in acyl CoA carboxylase and diacylglycerol acyl transporter activities has been reported in liver of a similar model system, the fructose-fed hamster [26]. The increase in muscle TG store could also be linked to impaired removal due to decreased tissue lipoprotein lipase activity in FRU rats [27].

A regulatory protein, called sterol regulatory element binding protein binds to sterol responsive elements found on multiple genes, and activates a cascade of enzymes involved in lipid biosynthesis pathway such as HMG-CoA reductase and fatty acid synthase. The activity of this protein in liver is reported to be enhanced in insulin resistant fructose-fed mice [28], and this explains the increased levels of cholesterol and fatty acids during fructose feeding.

The term “lipotoxicity” is used to refer to the condition involving accumulation of TG in insulin sensitive tissues associated with impairment in insulin action. An extramuscular defect of fatty acid metabolism could contribute to the intramyocellular TG accumulation leading to skeletal muscle lipotoxic effects in obesity and type 2 diabetes [29]. Increased delivery of TG to muscle tissues interferes with glucose utilization, through the principles of Randle cycle and impairs insulin action. Further excess production of metabolites from TG such as fatty acids, ceramides, and diacyl glycerol may enter deleterious nonoxidative pathways. Diacylglycerol accumulation specifically desensitizes insulin-stimulated glucose uptake in human muscle cell culture [30]. Accumulation of TG in skeletal muscle of fructose-treated rats has lipotoxic effect that contributes to insulin insensitivity.

The present study examined three indices of lipid peroxidation TBARS, LHP, and CD in skeletal muscle. The TBA test analyzes the end-products derived from hydroperoxide transformation, metabolism or decomposition. CDs are polyunsaturated molecules having alternate double bonds. They are formed at the onset of lipid peroxidation when polyunsaturated fatty acids are attacked by oxygen centred free radicals. CD are also linked to several steps of lipid peroxide degeneration. It is reported that about 30–35 percent of lipid peroxidation is actually detected by diene measurements [31]. LHP are measured by their ability to oxidize ferrous ion to ferric ion which depends not only on the rate of initiation of peroxidation but also their decomposition to other products. The procedure used, assesses the LHP, without interference from nonlipid hydroperoxides. Although the TBA test and CD measurement are very nonspecific, they can offer an empirical window on the complex process of lipid peroxidation [32]. Importantly, a more reliable marker of lipid peroxidation and oxidative injury 15-F_2_ isoprostane, a product of the nonenzymatic free-radical metabolism of arachidonic acid [33], has been shown to be elevated in plasma of FRU rats [34].

In fructose-fed rats free radical production can be enhanced during hyperinsulinemia and hyperglycemia by mechanisms such as autoxidation of glucose, enhanced glycation, and altered polyol pathway [35]. FFA could directly increase reactive oxygen species via peroxidation reactions and via mitochondrial production [36]. A study by Pennathur et al. [37] showed that rats with diet-induced hyperlipidemia without hyperglycemia fail to exhibit increased protein and lipid oxidation products in the retina. However Sies et al. [38] noted that hyperglycemia and/or hyperlipidemia can give rise to nutritional oxidative stress under postprandial conditions. Thus the presence of elevated lipid alone can cause oxidation of proteins and lipids that can be enhanced in the association with hyperglycemia. It is also important in this context to note that Harmon et al. [39] observed accumulation of TG in pancreas and insulin sensitive tissues in vivo only in presence of hyperglycemia.

Fructose itself can create oxidative stress by its metabolism. Depletion of ATP due to increased catabolism of fructose, down regulation of HMP shunt by fructose, increased aldehyde formation and reduced generation of reducing equivalents could be the contributing mechanisms [40]. Increase in protein carbonyl content and reduction in protein-thiols (P-SH) in FRU suggest protein oxidation. Ceriello et al. [41] reported that protein modification through increased free radical generation could reduce insulin activity.

CAR reduces intramitochondrial acyl-CoA/CoA ratio, promotes oxidative glucose utilization, lowers intracellular glucose levels, and improves insulin sensitivity [6, 8] and thereby prevents glucose toxicity.

Exogenous CAR alleviated the lipid accumulation in the skeletal muscle. The most obvious mechanism of TG lowering effect by CAR is its influence on the influx of fatty acids to the mitochondria. Intraperitoneal CAR restores plasma and liver lipids level in these animals [25]. CAR supplementation prevents fructose-induced cholesterol accumulation. This may depend on its TG lowering effect and its insulin sensitivity effects.

Owing to the amphiphilic nature and its interaction with the surface charges on the cell membrane, CAR can protect the cell, by transporting potentially toxic acyl compounds out of the mitochondria and the cell, enabling their subsequent excretion in urine [42].

CAR, by virtue of its ability to enhance ATP production, could bring a favorable metabolic environment and thus reduce oxidative stress. In addition, the vitamin C and methionine-sparing activity of CAR can reduce lipid peroxidation by maintaining the levels of other antioxidants like vitamin E and thiol groups. CAR also plays a role in chelating free Fe^2+^ ions and thereby limits Fenton-type reactions [43].

Repletion of GSH, the predominant NP-SH by CAR, may have an effect on insulin receptor gene activation. Efficient expression of insulin receptor gene requires certain transcription factors that are activated by GSH [44].

The present study shows that in fructose-fed rats, exogeneous CAR improves insulin sensitivity, reduces both lipo- and gluco-toxicity, and attenuates oxidative stress in skeletal muscle. The benefits could be attributed to its effects on glucose disposal, antioxidative mechanisms, and lipid profile. CAR levels are reported to be low in diabetic conditions [45] and CAR can be included as ingredient in nutritional supplements. The utility of CAR in this model should be of importance while considering the rising prevalence of insulin resistance. Additional studies on the effect of CA on the mitochondrial levels of FFAs and their metabolites and ATP synthesis in this animal model of fructose are to be undertaken in the near future.

## Figures and Tables

**Table 1:** Composition of diet (g/100 g).

Ingredients	Control diet	High-fructose diet

Corn starch	60	—
Fructose	—	60
Casein (fat free)	20	20
Methionine	0.7	0.7
Groundnut oil	5	5
Wheat bran	10.6	10.6
Salt mixture^♣^	3.5	3.5
Vitamin mixture^*^	0.2	0.2

^♣^ The composition of mineral mix (g/Kg)—MgSO_4_ · 7H_2_O-30.5; NaCl-65.2; KCl-105.7; KH_2_PO_4_-200.2; MgCO_3_-3.65; Mg(OH)_2_ · 3H_2_O-38.8; FeC_6_H_5_O_7_ · 5H_2_O-40.0; CaCO_3_-512.4; KI-0.8; NaF-0.9; CuSO_4_ · 5H_2_O-1.4; MnSO_4_-0.4, and CONH_3_-0.05.

* One kilogram of vitamin mix contained thiamine mononitrate, 3 g; riboflavin, 3 g; pyridoxine HCl, 3.5 g; nicotinamide, 15 g; d-calcium pantothenate, 8 g; folic acid,1 g; d-biotin, 0.1 g; cyanocobalamin, 5 mg; vitamin A acetate, 0.6 g; *α*-tocopherol acetate, 25 g, and choline chloride, 10 g.

**Table 2:** Levels of lipid hydroperoxides (LHP), thiobarbituric acid reactive substances (TBARS), conjugated dienes (CD), protein carbonyl, and aldehydes in skeletal muscles of control and experimental animals.

Parameters	CON	FRU	FRU + CAR	CON + CAR

LHP^(A)^	1.64 ± 0.14	2.27 ± 0.19^(a)^	1.74 ± 0.15^(b)^	1.55 ± 0.09
TBARS^(A)^	1.69 ± 0.11	2.20 ± 0.22^(a)^	1.67 ± 0.08^(b)^	1.52 ± 0.13
CD (A_233_/A_215_)	0.62 ± 0.06	0.92 ± 0.07^(a)^	0.66 ± 0.05^(b)^	0.60 ± 0.03
Protein carbonyl groups^(A)^	0.24 ± 0.02	0.31 ± 0.02^(a)^	0.25 ± 0.03^(b)^	0.22 ± 0.14
Aldehyde^(B)^	0.53 ± 0.04	0.61 ± 0.05^(a)^	0.50 ± 0.03^(b)^	0.49 ± 0.05

Values are means ± SD of 6 rats from each group. CON—control rats; FRU-fructose-fed rats; FRU + CAR—fructose fed rats treated with carnitine; CON + CAR—control rats treated with carnitine. ^(A)^ *μ*mol/mg protein; ^(B)^ *μ*mol quinine sulphate equivalents/g tissue.

^(a)^Significant as compared to CON (*P* < .05; ANOVA followed by DMRT).

^(b)^Significant as compared to FRU (*P* < .05; ANOVA followed by DMRT).

**Table 3:** Activities of enzymatic antioxidants in skeletal muscle of control and experimental animals.

Parameters	CON	FRU	FRU + CAR	CON + CAR

SOD (Units^(A)^)	3.39 ± 0.33	2.35 ± 0.20^(a)^	3.11 ± 0.13^(b)^	3.33 ± 0.22
CAT (Units^(B)^)	44.85 ± 3.74	31.36 ± 2.19^(a)^	42.15 ± 3.86^(b)^	46.18 ± 3.47
GPx (Units^(B)^)	5.25 ± 0.27	4.21 ± 0.42^(a)^	4.97 ± 0.47^(b)^	5.44 ± 0.41
GST (Units^(C)^)	4.15 ± 0.29	3.55 ± 0.33^(a)^	4.08 ± 0.18^(b)^	4.22 ± 0.18

Values are means ± SD of 6 rats from each group. CON-control rats; FRU—fructose-fed rats; FRU + CAR—fructose fed rats treated with carnitine; CON + CAR—control rats treated with carnitine. ^(a)^Significant as compared to CON (*P* < .05; ANOVA followed by DMRT). ^(b)^Significant as compared to FRU (*P* < .05; ANOVA followed by DMRT). ^(A)^amount of enzyme which gave 50% inhibition of nitro blue tetrazolium (NBT) reduction/mg protein; ^(B)^mol substrate/min/mg protein; ^(C)^nmoles of glutathione-1-chloro, 2,4-dinitrobenzene (CDNB) conjugate formed/min/mg protein.

**Table 4:** Concentrations of nonenzymatic antioxidants in skeletal muscle of control and experimental animals.

Parameters	CON	FRU	FRU + CAR	CON + CAR

NP-SH (Units^(A)^)	2.65 ± 0.10	1.03 ± 0.04^(a)^	2.46 ± 0.22^(b)^	2.72 ± 0.34
T-SH (Units^(B)^)	5.35 ± 0.43	2.71 ± 0.26^(a)^	5.40 ± 0.58^(b)^	6.00 ± 0.55
P-SH (Units^(B)^)	3.00 ± 0.35	1.68 ± 0.13^(a)^	2.93 ± 0.16^(b)^	2.80 ± 0.27
Vitamin C (Units^(A)^)	0.65 ± 0.07	0.39 ± 0.06^(a)^	0.59 ± 0.11^(b)^	0.66 ± 0.04
Vitamin E (Units^(A)^)	0.77 ± 0.09	0.43 ± 0.05^(a)^	0.67 ± 0.12^(b)^	0.79 ± 0.13

Values are means ± SD of 6 rats from each group. CON—control rats; FRU—fructose-fed rats; FRU + CAR—fructose-fed rats treated with carnitine; CON + CAR—control rats treated with carnitine. NP-SH—nonprotein thiol; T-SH—total thiol; P-SH—protein bound thiol; ^(a)^Significant as compared to CON (*P* < .05; ANOVA followed by DMRT). ^(b)^Significant as compared to FRU (*P* < .05; ANOVA followed by DMRT). ^(A)^(*μ*mol/mg protein); ^(B)^(*μ*g/mg protein).
